# A multicenter non-randomized, uncontrolled single arm trial for evaluation of the efficacy and the safety of the treatment with favipiravir for patients with severe fever with thrombocytopenia syndrome

**DOI:** 10.1371/journal.pntd.0009103

**Published:** 2021-02-22

**Authors:** Koichiro Suemori, Masayuki Saijo, Atsushi Yamanaka, Daisuke Himeji, Masafumi Kawamura, Takashi Haku, Michihiro Hidaka, Chinatsu Kamikokuryo, Yasuyuki Kakihana, Taichi Azuma, Katsuto Takenaka, Toru Takahashi, Akitsugu Furumoto, Toshiyuki Ishimaru, Masayuki Ishida, Masahiko Kaneko, Norimitsu Kadowaki, Kenichi Ikeda, Shigetoshi Sakabe, Tomohiro Taniguchi, Hiroki Ohge, Takeshi Kurosu, Tomoki Yoshikawa, Masayuki Shimojima, Masaki Yasukawa

**Affiliations:** 1 Department of Hematology, Clinical Immunology and Infectious Disease, Ehime University Graduate School of Medicine, Toon, Japan; 2 Department of Virology I, National Institute of Infectious Diseases, Shinjuku, Japan; 3 Department of Internal Medicine, Miyazaki Prefectural Miyazaki Hospital, Miyazaki, Japan; 4 Division of Internal Medicine, Kochi Prefectural Hata Kenmin Hospital, Sukumo, Kochi, Japan; 5 Department of Respiratory Medicine, Tokushima Prefectural Central Hospital, Tokushima, Japan; 6 Department of Hematology, National Hospital Organization Kumamoto Medical Center, Kumamoto, Japan; 7 Department of Emergency and Intensive Care Medicine, Kagoshima University Graduate School of Medical and Dental Sciences, Kagoshima, Kagoshima, Japan; 8 Department of Hematology, Yamaguchi Grand Medical Center, Hofu, Japan; 9 Division of Infectious Diseases, Department of Internal Medicine, Nagasaki Rosai Hospital, Nagasaki, Japan; 10 Department of Infectious Diseases, The Japanese Red Cross Fukuoka Hospital, Hakata, Japan; 11 Department of Infectious Diseases, Chikamori Hospital, Kochi, Kochi, Japan; 12 Department of Internal Medicine, Uwajima City Hospital, Uwajima, Japan; 13 Division of Hematology, Kagawa University Hospital, Kida, Japan; 14 Department of Internal Medicine, Kagoshima City Hospital, Kagoshima, Japan; 15 Department of Infectious Diseases, Ise Red Cross Hospital, Ise, Japan; 16 Division of General Internal Medicine & Infectious Diseases, Hiroshima Prefectural Hospital, Hiroshima, Japan; 17 Department of Infectious Diseases, Hiroshima University Hospital, Hiroshima, Japan; Seoul National University College of Medicine, REPUBLIC OF KOREA

## Abstract

Severe fever with thrombocytopenia syndrome (SFTS) is a bunyavirus infection with high mortality. Favipiravir has shown effectiveness in preventing and treating SFTS virus (SFTSV) infection in animal models. A multicenter non-randomized, uncontrolled single arm trial was conducted to collect data on the safety and the effectiveness of favipiravir in treatment of SFTS patients. All participants received favipiravir orally (first-day loading dose of 1800 mg twice a day followed by 800 mg twice a day for 7–14 days in total). SFTSV RT-PCR and biochemistry tests were performed at designated time points. Outcomes were 28-day mortality, clinical improvement, viral load evolution, and adverse events (AEs). Twenty-six patients were enrolled, of whom 23 were analyzed. Four of these 23 patients died of multi-organ failure within one week (28-day mortality rate: 17.3%). Oral favipiravir was well tolerated in the surviving patients. AEs (abnormal hepatic function and insomnia) occurred in about 20% of the patients. Clinical symptoms improved in all patients who survived from a median of day 2 to day10. SFTSV RNA levels in the patients who died were significantly higher than those in the survivors (p = 0.0029). No viral genomes were detectable in the surviving patients a median of 8 days after favipiravir administration. The 28-day mortality rate in this study was lower than those of the previous studies in Japan. The high frequency of hepatic dysfunction as an AE was observed. However, it was unclear whether this was merely a side effect of favipiravir, because liver disorders are commonly seen in SFTS patients. The results of this trial support the effectiveness of favipiravir for patients with SFTS.

## Author summary

Severe fever with thrombocytopenia syndrome (SFTS) is a tick-borne virus infection caused by Dabie bandavirus (formerly SFTS virus, SFTSV), which belongs to the *Bandavirus* genus of the *Phenuiviridae* family. The case fatality rate of patients with SFTS is high ranging from 16.2% to 47%. SFTS is endemic to East and Southeast Asia. Favipiravir, an antiviral agent with an inhibitory activity on the RNA-dependent RNA polymerase, inhibited replication of SFTSV *in vitro* and *in vivo*. It was suggested that favipiravir treatment lowered the case fatality rate of patients with SFTS by approximately 10% in comparison with those reported so far through epidemiological survey in Japan. People living in the SFTS-endemic regions can not escape from the risk of being infected with SFTSV. Favipiravir might be an effective drug for treatment of patients with SFTS and reduces the mortality and morbidity of patients with SFTS.

## Introduction

Severe fever with thrombocytopenia syndrome (SFTS) is a tick-borne virus infection caused by Dabie bandavirus (formerly SFTS virus, SFTSV), which belongs to the *Bandavirus* genus (formerly *Phlebovirus* genus) of the *Phenuiviridae* family (formerly *Bunyaviridae* family). SFTS has been identified in China, South Korea, and Japan since the early 2010s [1–4]. The virus name SFTSV is used in this article. SFTSV is maintained in nature between several species of tick and both wild and domestic animals [5]. Humans are usually infected with SFTSV through tick bites. Some patients with SFTS were infected with the virus through close contact with sick domestic animals such as cats infected with SFTSV [6]. SFTS patients were also reported in Vietnam and Taiwan [7–9].

The typical clinical manifestations are fever, fatigue, gastrointestinal symptoms, hemorrhagic tendency, and deterioration of consciousness. Leukocytopenia and thrombocytopenia appear in total blood cell counts, and levels of aspartate aminotransferase (AST), alanine aminotransferase (ALT), creatinine kinase (CK), and lactate dehydrogenase (LDH) are elevated in serum chemistry [1,4,10]. Patients with severe SFTS progress to multiple organ dysfunction, hemophagocytosis, and disseminated intravascular coagulation, and the majority of such cases prove fatal within 7–14 days after onset. Case fatality rates (CFRs) among SFTS patients were from 16.2%, 27–47%, and 27–31%, in China, South Korea, and Japan, respectively [11–14]. Older age and a high viral load are critical risk factors for SFTS mortality [13,15]. It is of note that the CFR in Japan was reported to be 31% [11] and 27% [12]. The aim of this study was to evaluate an efficacy and a safety of antiviral therapy with favipiravir (6-fluoro-3-hydroxy-2-pyrazinecarboxamide) for patients with SFTS in Japan.

Ribavirin as an antiviral therapy and steroid pulse therapy and plasma exchange as supportive therapies have been applied to SFTS patients, although their efficacy remains limited [13,16–18]. Favipiravir, an RNA-dependent RNA polymerase inhibitor, which has been approved for influenza pandemic preparedness in Japan [19], showed efficacy for the prevention and treatment of SFTSV infection in animal models [20–22]. While interferon-α receptor knockout (IFNAR-KO) mice were infected with 1.0 × 10^6^ 50% tissue culture infective dose (TCID_50_) of SFTSV subcutaneously, all mice died within 5–7 days after infection, all the SFTSV-infected IFNAR−KO mice survived when the mice were administered with favipiravir within 3 days post infection [20,21]. The viremia level of the SFTSV-infected mice that were treated with favipiravir at the dose of 300 mg/kg/day became undetectable by highly sensitive SFTSV genome detection with quantitative real-time reverse transcription polymerase chain reaction (qRT-PCR) [15], even at 2 days after the initiation of favipiravir treatment [20]. Hamsters devoid of functional STAT2 (STAT2 knockout (KO) hamsters) are reported to be highly susceptible to as few as 10 plaque forming unit (PFU) of SFTSV. The hamsters succumb to death within 5 to 6 days after subcutaneous challenge. It was reported that SFTSV infection in STAT2 knockout (KO) hamsters infected with SFTSV were protected from lethal infection by the treatment with favipiravir [22].

Because SFTSV is circulating in nature, we can not escape from the risk of being infected with SFTSV. Development of therapeutics is required for patients with SFTS [23]. In the present study, a prospective, open-label, single arm, multi-center study was conducted to evaluate the efficacy and the safety of favipiravir in the treatment of SFTS patients in Japan.

## Patients and methods

### Ethics statement

The study protocol, informed consent form, and all other required documents were reviewed and approved by an Independent Ethics Committee for each study site before initiation of the study at that site. The identification number for approval issued was #684 from the Research and Ethics Committee of the National Institute of Infectious Diseases (NIID). The identification numbers for approval issued from the institutional ethical committee of each hospital are #1603038 and #1708017 (Ehime University Graduate School of Medicine), #16–09 (Miyazaki Prefectural Miyazaki Hospital), #H28-24 (Kochi Prefectural Hata Kenmin Hospital), #16–1 (Tokushima Prefectural Central Hospital), #674 (National Hospital Organization Kumamoto Medical Center), #2017–017 (Yamaguchi Grand Medical Center), #29010 (Nagasaki Rosai Hospital), #374 (The Japanese Red Cross Fukuoka Hospital), #212 (Chikamori Hospital), #167–101 (Uwajima City Hospital), #H28-037 and #H29-136 (Kagawa University Hospital), #2017–36 (Kagoshima City Hospital), #29–53 (Ise Red Cross Hospital), #2016–001 and #H28-21 (Hiroshima Prefectural Hospital), #170200 (Kagoshima University Graduate School of Medical and Dental Sciences), and #C-93 and #C-210-2 (Hiroshima University Hospital). This study was conducted in accordance with Japanese Ethical Guidelines for Medical and Health Research Involving Human Subjects (December 2014). These guidelines are consistent with the World Medical Assembly Declaration of Helsinki. Written informed consent was obtained from each subject or advocate for the subject (spouse or lineal relatives) before any study-specific procedures were performed.

### Study design

The primary endpoint was overall survival (OS), which was defined as the mortality rate during the 28-day period after the introduction of favipiravir, in the enrolled cohort. The secondary endpoints were the time until SFTSV became undetectable in peripheral blood as measured by virus isolation assay [4], changes in the SFTSV load determined by quantitative real-time reverse transcription polymerase chain reaction (qRT-PCR) [15], and the time until alleviation of clinical symptoms and normalisation of laboratory parameters. This study was conducted during two periods, April to December 2016 and September 2017 to July 2018, mainly in western Japan. Microbiological tests including blood, urine, and throat cultures were performed routinely depend on the patients’ conditions by the attending physicians. However, it was of note that the microbiological tests except for SFTS-associated tests were not included in the study protocol.

### Participants

Participants were patients diagnosed as or strongly suspected of having SFTS and who satisfied both the inclusion and exclusion criteria.

Inclusion criteria were as follows: 1) patients aged 20 years or older, 2) patients diagnosed as having SFTS with SFTSV genome positive by both the conventional RT-PCR and the qRT-PCR [15] before favipiravir-treatment initiation, 3) patients strongly suspected of having SFTS on the basis of findings such as a tick bite, temperature ≥38°C, WBC count <4,000/μL, platelet count <100,000/μL, gastrointestinal tract symptoms (nausea, vomiting, abdominal pain, diarrhea, melena), and occult hematuria, and 4) patients who understood the requirements of the study and provided written informed consent prior to undergoing any study-related procedures.

Exclusion criteria were as follows: 1) patients whose clinical symptoms had already improved or whose SFTSV load in peripheral blood had already been in the decreasing phase, 2) patients in whom more than seven days had passed from onset of symptoms, 3) patients who had been treated for Grade C-equivalent liver impairment based on the Child-Pugh classification [24], 4) patients who were pregnant or had a positive pregnancy test prior to initial dosing, 5) patients who had difficulty in adhering to very effective measures of contraception with their partners during favipiravir treatment and for seven days following the last dose of favipiravir, 6) patients with hereditary xanthinuria, a history of hypouricemia (urea acid <1 mg/dL), or xanthine calculi in the urinary tract, 7) patients who had a history of hypersensitivity to favipiravir, 8) patients who were deemed ineligible for any reason by the principal investigator or subinvestigator, and 9) patients, who were treated with ribavirin or drugs and medical equipment under development.

### Rank classification of clinical symptoms and laboratory findings

The severity of clinical symptoms and laboratory findings were classified in accordance with a pre-specified rank for making it available to assess the clinical course of the SFTS patients treated with favipiravir in terms of severity and outcome (S1 Table). In brief, the classifications were as follows, Rank 1, normal level; Rank 2, mild level; Rank 3, moderate level; and Rank 4, severe level.

### Virological test for SFTSV detection and isolation

SFTSV infection was confirmed using the conventional RT-PCR at the local public health institutions and/or the NIID, as described previously [15]. Quantitative real-time RT-PCR was also performed on peripheral blood collected throughout the study (every 3 days from the day of fever onset until day 19 of disease) at the NIID. Virus culture was performed to evaluate the day on which infectious SFTSV became undetectable. Peripheral blood samples were collected throughout the study (every 3 days from the day of fever onset until day 10 of disease) for virus culture testing.

### Usage, dose, and administration period of favipiravir

Patients were administered favipiravir orally at a loading dose of 1,800 mg twice a day on the first day followed by 800 mg twice a day for 7–14 days in total. If the patients could not take favipiravir tablets orally, the patients were administered the tablet in a simple suspension form through a gastric tube.

### Safety assessments

Adverse events (AEs) were evaluated by attending physicians until 28 days after the start of favipiravir administration, regardless of whether they had any causal relationship with the favipiravir. The severity of AEs was classified into the following three grades in accordance with a notice from the Ministry of Health, Labour and Welfare of Japan (MHLW) [25]: Grade 1: AE mild, allowing the study to be continued and not interfering with daily life; Grade 2: AE moderate, causing the study to disturb daily life but not affecting function; Grade 3: AE moderate, causing the study to disturb daily life and possibly leading to permanent malfunction requiring medical treatment; or severe AE: fatal, life-threatening, extension of hospitalisation, permanent or marked disability and malfunction, occurrence of birth defects and medically important abnormality. Adverse reactions (ARs) were evaluated by attending physicians based on the pharmaceutical document providing information on the side effects of favipiravir.

### Data management

Demographic patient data were recorded by the principal investigator or subinvestigator on a pre-designed case report form. The anonymized demographic data included subject ID, date of provision of informed consent, date of birth, sex, race and nationality, weight, height, medical history, allergies, date of fever onset, diagnosis, and pregnancy status. These data were collected before favipiravir-treatment initiation. The demographic data together with data from laboratory sample report forms containing sample ID, sample type, collection date, date of symptom onset, and SFTSV RT-PCR results were entered in a database (Excel, Microsoft) at the Center for Clinical Research Data and Biostatistics, Ehime University Hospital, Toon, Ehime, Japan. Data recorded were checked for consistency with the source data by the research staff. Data were managed according to standard operating procedures established in advance.

### Statistical analysis

All categorical variables are presented with absolute frequency counts and percentages. Verification of all analyses and summaries were conducted using SAS software Version 9.4 (SAS Institute Inc., Cary, NC). The characteristics of patients who survived were compared with those of patients who died using the Student *t*-test or Mann-Whitney U test for continuous variables and Fisher’s exact test for categorical variables. Survival time for SFTS patients was plotted using the Kaplan-Meier method. The difference in the survival rates between patients with higher viremia and those with relatively lower viremia was statistically evaluated with Log-rank test. Number of events, number censored, median time to event, and its 95% confidence interval are presented in the analysis table. All statistical testing was two-sided at a type-I error rate of 0.05.

### Monitoring of favipiravir blood levels

Blood samples were taken post-dose (1 to 2 hours after dosing in the morning) on day 1 and pre-dose (just prior to dosing in the morning) and post-dose (1 to 2 hours after dosing in the morning) on days 4 and 7 or 10. Model-based pharmacokinetic analysis was conducted to estimate the time course of favipiravir blood levels in the subjects mainly using Phoenix WinNonlin version 6.3 (Certara LP, Princeton, NJ). The blood samples for monitoring of favipiravir blood levels were taken only from the patients, who participated in this study during the first period of April—December 2016.

## Results

### Characteristics and outcome of the participants

**Fig 1:**
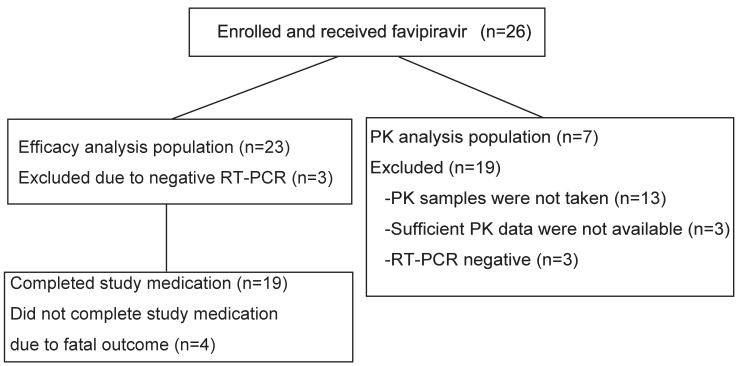
Patient flow diagram. Twenty-six patients were enrolled but 3 were excluded from the favipiravir efficacy analyses because of negative RT-PCR for SFTSV genome amplification (left column). Pharmacokinetic (PK) analyses were done using seven patients (right column).

Twenty-six patients aged between 42 and 91 years were enrolled (Fig 1).

**Fig 2:**
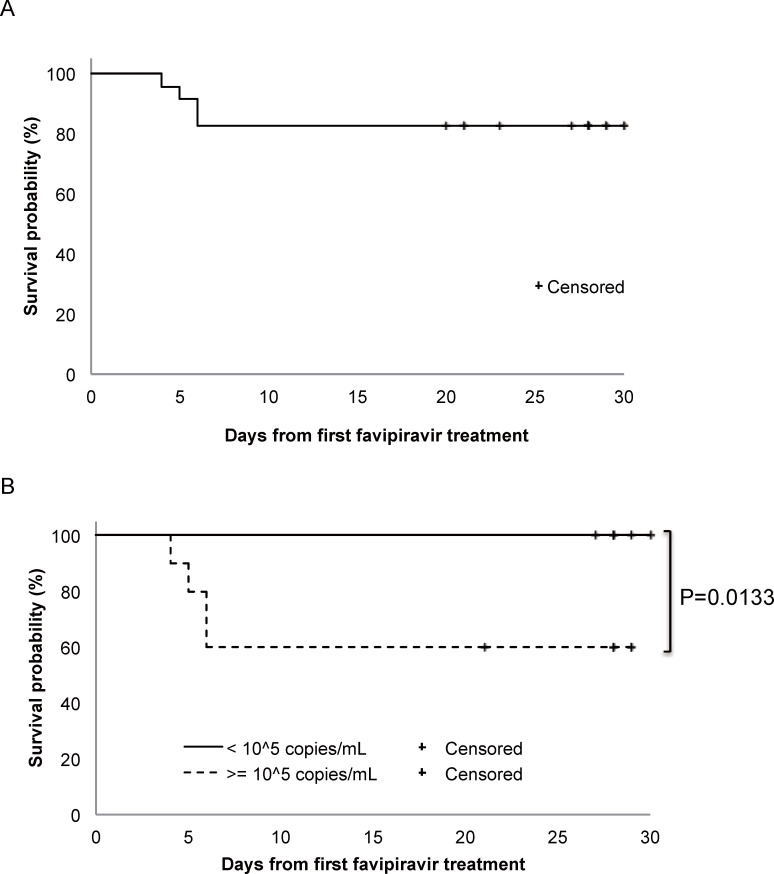
Survival rate in the 23 SFTS patients who received favipiravir. Overall survival rate in the 23 SFTS patients who received favipiravir is shown based on the survival time plotted using the Kaplan-Meier method (A). Survival probabilities of patients with higher SFTSV viremia level (≥ 1 × 10^5^ copies/mL, dotted line, n = 10) and that of patients with lower SFTSV viremia level (< 1 × 10^5^ copies/mL, solid line, n = 13) are shown (B).

Three patients who were suspected of having SFTS were initially included but were then ultimately excluded because their blood samples were negative for SFTSV by conventional RT-PCR. Therefore, 23 patients were enrolled for further analyses. Nineteen patients (Non-fatal group) completed the study medication course, whereas 4 patients, who died (Fatal group), could not. The four who died did so of multiple organ failure within 6 days, resulting in an overall survival (OS) rate of 82.6% (Fig 2A).

The basic characteristics of the patients are summarised (Table 1).

**Table 1:** Basic characteristics of the 23 SFTS patients who received favipiravir.

Categories	Subcategories	Overall (n = 23)	Groups		p-Value
			Fatal (n = 4)	Non-fatal (n = 19)	
Gender	Male	14 (60.9%)	1 (25%)	13 (68.4%)	0.2601*
	Female	9 (39.1%)	3 (75%)	6 (31.6%)	
Age (years)	Median (IQR)	71.0 (65, 81)	73.5 (63.5)	71.0 (65, 81)	0.9031^†^
	< 60	2 (8.7%)	0 (0%)	2 (10.5%)	0.7123*
	60–79	14 (60.9%)	2 (50%)	12 (63.2%)	
	≥ 80	7 (30.4%)	2 (50%)	5 (26.3%)	
Weight (kg) ^Ω^	Median (IQR)	57.3 (50.0, 69.4)	47.5 (45.7, 58.7)	59.1 (50.4, 71.0)	0.1230^†^
BMI (kg/m^2^)^Ω^	Median (IQR)	22.2 (19.9, 25.1)	21.4 (20.4, 23.4)	23.1 (19.7, 26.0)	0.5429^†^
Time from onset to favipiravir initiation (days)	Median (IQR)	4 (3, 5)	4.5 (3 5.5)	4.0 (3, 5)	0.7730^†^
Underlying disease	Diabetes mellitus	4 (17.4%)	0 (0%)	4 (21.1%)	1.00*
	Cancer	1 (4.4%)	0 (0%)	1 (5.3%)	1.00*
	Hypertension	12 (52.2%)	0 (0%)	12 (63.2%)	0.0373*
	Hyperlipidaemia	5 (21.7%)	0 (0%)	5 (26.3%)	0.5392*

* Fisher’s exact test.

† Mann-Whitney U test.

Ω measured before initiation of favipiravir treatment.

n = number, IQR = interquartile range, BMI = body mass index.

The median age of the patients was 71 years, and 14 of them were men. There were no significant differences between the Fatal and Non-fatal groups in terms of age, weight, body mass index, and time from disease onset until favipiravir initiation. Underlying diseases were reported in 19 of the study patients (82.6%): hypertension in twelve, hyperlipidemia in five, diabetes mellitus in four, and cancer in one. There was a statistical difference in having hypertension between the two groups.

### Clinical characteristics of the patients on admission

The signs and symptoms evident on admission did not significantly differ between the Fatal and Non-fatal groups (S2 Table). Laboratory findings on admission are summarised in Table 2. The viral genome load was the only factor that differed significantly between the Fatal and Non-fatal groups. The viral genome loads in the Fatal group patients was 3.16 × 10^6^ copies/mL and was significantly higher than that of 5.01 × 10^4^ copies/mL in the Non-fatal group (p = 0.0029). The rank classification of diarrhea and the serum LDH level on admission differed significantly between the two groups (p = 0.03 and p = 0.02, respectively) among the symptoms and the laboratory parameters evaluated (Table 2).

**Table 2:** Laboratory findings on admission.

Categories	Normal range*	Overall [Mean (SD)]	Groups		p-Value†
			Fatal (n = 4) [Mean (SD)]	Non-fatal (n = 19) [Mean (SD)]	
WBC (/mm^3^)	3,300–8,600	2,737 (2,527)	1,337 (457)	3,032 (2691)	0.2312
Hb (g/dL)	M: 13.7–16.8 F: 11.6–14.8	14.2 (2.2)	14.2 (1.4)	14.2 (2.4)	0.9786
Plt (×10^4^/ mm^3^)	15.8–34.8	5.2 (1.4)	5.2 (1.2)	5.2 (1.5)	0. 9909
Albumin (g/dL)	4.1–5.1	3.1 (0.6)	3.2 (1.0)	3.1 (0.5)	0.6138
AST (U/L)	13–30	278 (193)	367 (161)	259 (197)	0.3189
ALT (U/L)	M: 10–42 F: 7–23	110 (80.9)	154 (97.7)	101 (76.7)	0.2422
LDH (U/L)	124–222	890 (431)	926 (123)	882 (473)	0.8541
CK (U/L)	M: 59–248 F: 41–153	1,897 (2,430)	1,105 (1,001)	2,064 (2,623)	0.4859
Cr (mg/dL)	M: 0.65–0.07 F: 0.46–0.79	1.07 (0.76)	0.79 (0.19)	1.13 (0.82)	0.4254
BUN (mg/dL)	8–20	22.4 (11.2)	17.7 (5.9)	23.3 (11.9)	0.3768
CRP (mg/dL)	0.00–0.14	0.9 (1.1)	1.0 (1.4)	0.9 (1.0)	0.9435
Ferritin (ng/mL)	M: 18.6–261 F: 4.0–64.2	8,156 (112 340)	16,367 (23 629)	6,428 (6 574)	0.1094
Viral genome load (log_10_ copies/mL)		5.0 (1.2)	6.5 (1.5)	4.7 (0.9)	0.0029

* Japanese Committee For Clinical Laboratory Standards, JCCLS

† Student *t*-test.

The rank classification of clinical symptoms and laboratory findings on admission are summarised (Table 3).

**Table 3:** Rank classification of clinical symptoms and laboratory findings on admission.

Clinical symptoms and laboratory findings	Number of patients evaluated	Rank	Overall	Groups		p-Value*
				Fatal	Non-fatal	
Body temperature	23	1	8 (34.8%)	1/4 (25.0%)	7/19 (36.8%)	0.66
		2	8 (34.8%)	1/4 (25.0%)	7/19 (36.8%)	
		3	5 (21.7%)	1/4 (25.0%)	4/19 (21.1%)	
		4	2 (8.7%)	1/4 (25.0%)	1/19 (5.3%)	
Headache	19	1	13 (68.4%)	1/3 (33.3%)	12/16 (75.0%)	0.09
		2	5 (26.3%)	1/3 (33.3%)	4/16 (25.0%)	
		3	1 (5.3%)	1/3 (33.3%)	0/16 (0%)	
		4	0 (0%)	0/3 (0%)	0/16 (0%)	
Body aches and pains	18	1	12 (66.7%)	2/3 (66.7%)	10/15 (66.7%)	1.00
		2	2 (11.1%)	0/3 (0%)	2/15 (13.3%)	
		3	4 (22.2%)	1/3 (33.3%)	3/15 (20.0%)	
		4	0 (0%)	0/3 (0%)	0/15 (0%)	
Vomiting	21	1	17 (81.0%)	4/4 (100%)	13/17 (76.5%)	0.55
		2	4 (19.0%)	0/4 (0%)	4/17 (23.5%)	
		3	0 (0%)	0/4 (0%)	0/17 (0%)	
		4	0 (0%)	0/4 (0%)	0/17 (0%)	
Abdominal pain	18	1	12 (66.7%)	1/3 (33.3%)	11/15 (73.3%)	0.10
		2	5 (27.8%)	1/3 (33.3%)	4/15 (26.7%)	
		3	1 (5.6%)	1/3 (33.3%)	0/15 (0%)	
		4	0 (0%)	0/3 (0%)	0/15 (0%)	
Diarrhea	20	1	10 (50.0%)	0/3 (0%)	10/17 (58.8%)	0.03
		2	7 (35.0%)	1/3 (33.3%)	6/17 (35.3%)	
		3	2 (10.0%)	1/3 (33.3%)	1/17 (5.9%)	
		4	1 (5.0%)	1/3 (33.3%)	0/17 (0%)	
Dyspnea	18	1	13 (72.2%)	2/2 (100%)	11/16 (68.8%)	1.00
		2	4 (22.2%)	0/2 (0%)	4/16 (25.0%)	
		3	1 (5.6%)	0/2 (0%)	1/16 (6.3%)	
		4	0 (0%)	0/2 (0%)	0/16 (0%)	
Hemorrhage	22	1	18 (81.8%)	4/4 (100%)	14/18 (77.8%)	1.00
		2	1 (4.5%)	0/4 (0%)	1/18 (5.6%)	
		3	1 (4.5%)	0/4 (0%)	1/18 (5.6%)	
		4	2 (9.1%)	0/4 (0%)	2/18 (11.1%)	
Disorientation	21	1	9 (42.9%)	1/4 (25.0%)	8/17 (47.1%)	0.53
		2	9 (42.9%)	2/4 (50.0%)	7/17 (41.2%)	
		3	2 (9.5%)	1/4 (25.0%)	1/17 (5.9%)	
		4	1 (4.8%)	0/4 (0%)	1/17 (5.9%)	
WBC	23	1	2 (8.7%)	0/4 (0%)	2/19 (10.5%)	0.21
		2	9 (39.1%)	0/4 (0%)	9/19 (47.4%)	
		3	10 (43.5%)	3/4 (75.0%)	7/19 (36.8%)	
		4	2 (8.7%)	1/4 (25.0%)	1/19 (5.3%)	
Plt	23	1	0 (0%)	0/4 (0%)	0/19 (0%)	0.66
		2	1 (4.3%)	0/4 (0%)	1/19 (5.3%)	
		3	12 (52.2%)	3/4 (75.0%)	9/19 (47.4%)	
		4	10 (43.5%)	1/4 (25.0%)	9/19 (47.4%)	
AST	23	1	0 (0%)	0 /4 (0%)	0/19 (0%)	0.69
		2	4 (17.4%)	0/4 (0%)	4/19 (21.1%)	
		3	2 (8.7%)	0/4 (0%)	2/19 (10.5%)	
		4	17 (73.9%)	4/4 (100%)	13/19 (68.4%)	
ALT	23	1	4 (17.4%)	0/4 (0%)	4/19 (21.1%)	0.09
		2	8 (34.8%)	0/4 (0%)	8/19 (42.1%)	
		3	5 (21.7%)	2/4 (50.0%)	3/19 (15.8%)	
		4	6 (26.1%)	2/4 (50.0%)	4/19 (21.1%)	
LDH	23	1	0 (0%)	0/4 (0%)	0/19 (0%)	0.02
		2	8 (34.8%)	0/4 (0%)	8/19 (42.1%)	
		3	9 (39.1%)	4/4 (100%)	5/19 (26.3%)	
		4	6 (26.1%)	0/4 (0%)	6/19 (31.6%)	
CK	23	1	3 (13.0%)	0/4 (0%)	3/19 (15.8%)	1.00
		2	3 (13.0%)	1/4 (25.0%)	2/19 (10.5%)	
		3	6 (26.1%)	1/4 (25.0%)	5/19 (26.3%)	
		4	11 (47.8%)	2/4 (50.0%)	9/19 (47.4%)	
Viral load	23	1	0 (0%)	0/4 (0%)	0/19 (0%)	0.053
		2	5 (23%)	0/4 (0%)	5/19 (26.3%)	
		3	13 (56.5%)	1/4 (25.0%)	12/19 (63.2%)	
		4	5 (21.7%)	3/4 (75.0%)	2/19 (10.5%)	

* Fisher’s exact test.

WBC = white blood cell count, Plt = platelet count, AST = aspartate transaminase, ALT = alanine transaminase, LDH = lactate dehydrogenase, CK = creatine kinase

The time to resolution (rank 0) of clinical symptoms and laboratory findings in the Non-fatal group is shown (Table 4).

**Table 4:** Time until resolution of clinical symptoms and normalization of laboratory parameters in the Non-fatal group.

Clinical symptoms and laboratory findings	Number evaluated	Median days	95% CI
Body temperature	19	4.0	2.0, 8.0
Headache	18	4.0	2.0, 8.0
Body aches and muscle pains	18	8.0	2.0, 13.0
Vomiting	19	2.0	2.0, 8.0
Abdominal pain	18	2.5	2.0, 13.0
Diarrhea	19	5.0	2.0, 12.0
Dyspnea	17	10.0	4.0, 21.0
Hemorrhage	19	10.0	5.0, 22.0
Disorientation	19	11.0	3.0, 14.0
Plt	19	10.0	8.0, 14.0
WBC	19	11.0	7.0, 28.0
AST	19	23.0	14.0,
ALT	19	21.0	20.0, 26.0
LDH	19	28.0	22.0,
CK	19	10.5	7.0, 14.0
Viral load	19	7.0	6.0, 9.0

CI = confidence interval, Plt = platelet count, WBC = white blood cell count, AST = aspartate transaminase, ALT = alanine transaminase, LDH = lactate dehydrogenase, CK = creatine kinase.

Among the clinical symptoms, vomiting and abdominal pain improved early at a median of 2 and 2.5 days, respectively. However, improvement of dyspnea, hemorrhage, and disorientation occurred later, at a median of 10, 10, and 11 days, respectively. Among laboratory parameters, the WBC and platelet counts and CK level improved at a median of 10, 11, and 10.5 days, respectively. The improvements in AST, ALT, and LDH levels occurred much later at a median of 23, 21, and 28 days, respectively. SFTSV genome became negative in all the patients in the Non-fatal group by 16 days with a median of 13 days.

Each of the combination therapies offered to the patients participated was not initiated in relation with favipiravir treatment, but was initiated based on each patient’s conditions by the attending physicians. There was no significant difference in the combination therapies performed for each patient based on the attending physicians’ decision between the Fatal and Non-fatal groups in terms of combination therapy, except for the incidence of mechanical ventilation (Table 5). Corticosteroid therapy including steroid pulse therapy was performed for 11 of 23 patients targeting a pathophysiology of hemophagocytic syndrome-associated cytokine storm, which is one of the main factors for poor prognosis of SFTS patients [4,26–29], although the steroid therapy had not been confirmed to be effective for SFTS patients. In the present study, there was no significant relationship between patients who received corticosteroid therapy and those who received mechanical ventilation respiratory support therapy.

**Table 5:** Combination therapy performed for enrolled patients with SFTS.

Treatment categories	Overall (n = 23)	Groups		p-Value*
		Fatal (n = 4)	Non-fatal (n = 19)	
Corticosteroids	11 (48%)	3 (75%)	8 (42%)	0.32
Steroid pulse therapy	8 (35%)	2 (50%)	6 (32%)	0.59
Antimicrobial agents				
Quinolone	10 (43%)	1 (25%)	9 (47%)	0.6
Tetracycline	5 (22%)	1 (25%)	4 (21%)	1.00
Glycopeptide	2 (9%)	0 (0%)	2 (11%)	1.00
Other	1 (4%)	0 (0%)	1 (5%)	1.00
Candin	6 (26%)	0 (0%)	6 (32%)	0.54
Blood products transfused				
Platelets	8 (35%)	3 (75%)	5 (26%)	0.10
Red blood cells	2 (9%)	1 (25%)	1 (5%)	0.32
Fresh frozen plasma	2 (9%)	0 (0%)	2 (11%)	1.00
Immunoglobulin	4 (17%)	1 (25%)	3 (16%)	1.00
Thrombomodulin	3 (13%)	1 (25%)	2 (11%)	0.45
Blood purification therapy	3 (13%)	1 (25%)	2 (11%)	0.45
Mechanical ventilation	6 (26%)	3 (75%)	3 (16%)	0.04

* Fisher’s exact test.

### Viremia level and virus isolation in SFTS patients treated with favipiravir

**Fig 3:**
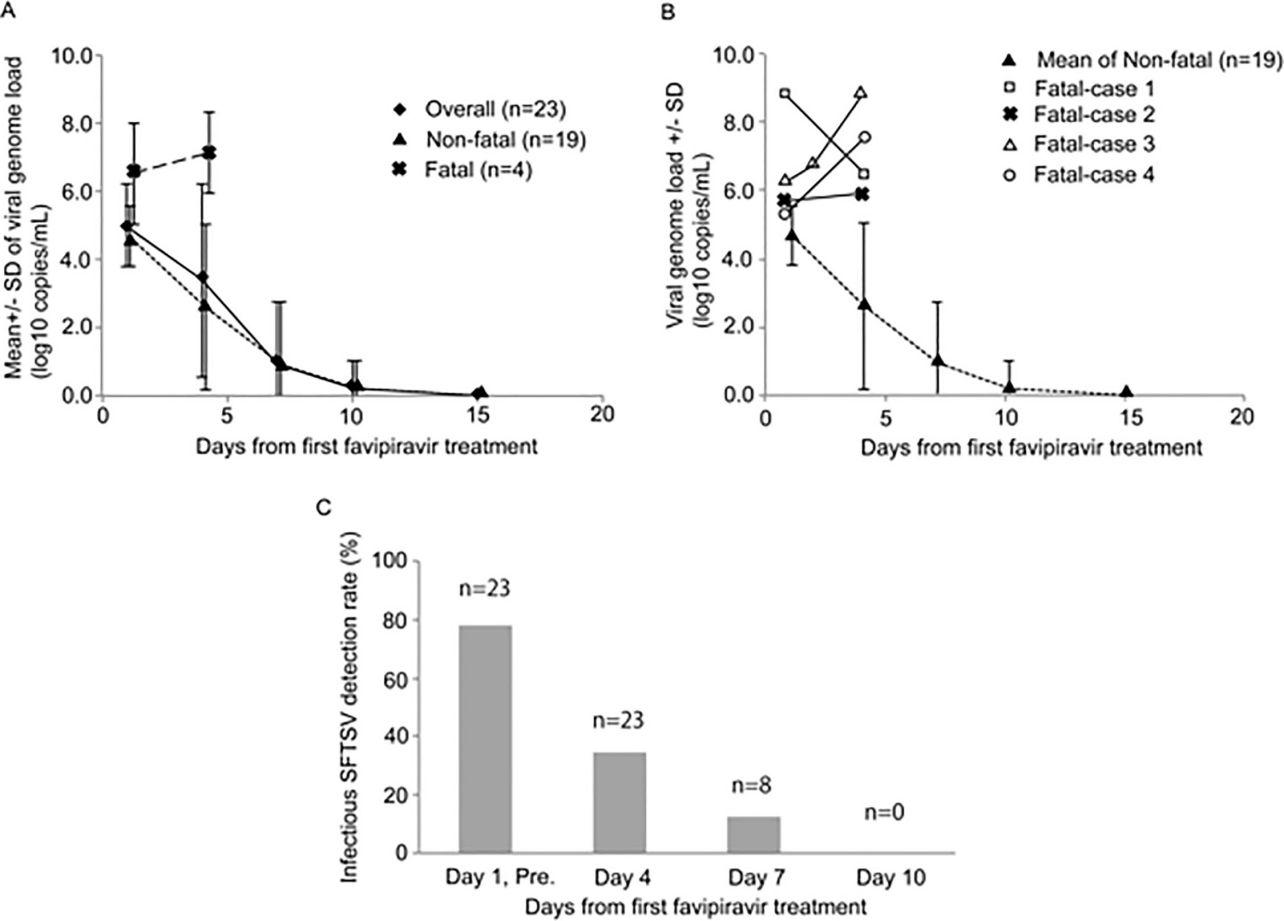
Transition in the amount of SFTSV in overall patients, the Non-fatal group, and Fatal group. Transition in the amount of SFTSV in overall patients, the Non-fatal group, and Fatal group as determined by real-time RT-PCR is shown (A). The genome levels in each patient who died of SFTS are also plotted as well as those of the Non-fatal group (B). The SFTSV survival rate in terms of the isolation test for SFTSV is shown (C). “n” indicates the number of serum samples tested for virus isolation. When a serum sample became negative for SFTSV genome with qRT-PCR in one patient, the serum samples collected from the patients after the day on which it became SFTSV genome negative were not always subjected for virus isolation. Therefore, the numbers of serum samples subjected for virus isolation collected on Day 1, Day 4, Day 7, and Day 10 became 23, 23, 8, and 3, respectively. The rates of positive virus isolation were 78% (18/23), 35% (8/23), 12% (1/8), and 0% (0/3) on Day 1 and pre-dose (pre.), Day 4, Day 7, and Day10, respectively.

The viral load decreased day by day in the patients overall (Fig 3A). However, the course of the SFTSV load in the Fatal group was apparently different from that in the Non-fatal group. The sequential viral load in Fatal group patients is shown (Fig 3B). The rates of positive virus isolation decreased as the disease course progressed (Fig 3C). Viral clearances evaluated with virus isolation and qRT-PCR in the Non-fatal group were obtained after a median of 8 days and 13 days, respectively (Table 4).

### Adverse events and reactions

AEs and ARs as judged by the attending physicians are summarised (Table 6).

**Table 6:** Summary of adverse events and adverse reactions.

Categories	Efficacy population (n = 23)		
	n	%	95% CI
Any adverse event (AE)	20	87.0	66.4, 97.2
Grade 1	14	60.9	38.5, 80.3
Grade 2	7	30.4	13.2, 52.9
Grade 3	7	30.4	13.2, 52.9
Serious AE	4	17.4	5.0, 38.8
Any adverse reaction (AR)	13	56.5	34.5, 76.8
Grade 1	8	34.8	16.4, 57.3
Grade 2	6	26.1	10.2, 48.4
Grade 3	2	8.7	1.1, 28.0
Serious AR*	1	4.3	0.1, 21.9
AE leading to treatment discontinuation (HLGT/PT)	4	17.4	5.0, 38.8
Fatal outcomes	2	8.7	1.1, 28.0
Death	2	8.7	1.1, 28.0
General system disorders NEC	1	4.3	0.1, 21.9
Condition aggravated	1	4.3	0.1, 21.9
Hepatic and hepatobiliary disorders	2	8.7	1.1, 28.0
Hepatic function abnormal	2	8.7	1.1, 28.0
Renal disorders (excl. nephropathies)	1	4.3	0.1, 21.9
Acute kidney injury	1	4.3	0.1, 21.9
AE occurring in 20% or more (HLGT/PT)			
Epidermal and dermal conditions	6	26.1	10.2, 48.4
Erythema	2	8.7	1.1, 28.0
Generalised erythema	1	4.3	0.1, 21.9
Pruritus	1	4.3	0.1, 21.9
Rash	3	13	2.8, 33.6
Hepatic and hepatobiliary disorders	8	34.8	16.4, 57.3
Hepatic function abnormal	5	21.7	7.5, 43.7
Hyperbilirubinemia	2	8.7	1.1, 28.0
Liver disorder	1	4.3	0.1, 21.9
Sleep disorders and disturbances	5	21.7	7.5, 43.7
Insomnia	5	21.7	7.5, 43.7

* Preferred term, Seizure

** System Organ Class, Hepatobiliary disorders

n = number, CI = confidence interval, HLGT = High Level Group Term, PT = Preferred Term, NEC = Not Elsewhere Classified.

The frequency of AEs and ARs was 87% and 56.5%, respectively. Serious AEs occurred in four patients, and the serious ARs were death and convulsion. AEs leading to treatment discontinuation occurred in the four patients who died. Hepatic dysfunction and insomnia were the AEs that occurred in 20% or more of the patients.

### Viremia level and survival probability

Survival probability in relation to viral load at the start of favipiravir administration was statistically significant with a virus genome load of 1 × 10^5^ copies/mL (p = 0.01) (Fig 2B). The SFTSV survival rate in terms of the isolation test for SFTSV decreased as the course of favipiravir treatment progressed. The CFRs in SFTS patients with SFTS ≥ 1 × 10^5^ copies/mL of SFTSV genome viremia and those with SFTS < 1 × 10^5^ copies/mL of SFTSV genome viremia in the present study was 40% (4/10) and 0% (0/13), respectively, with a significant difference (P-value of 0.013, Log-rank test). The patients in the Fatal group died within 6 days from the favipiravir treatment initiation (Fig 2B).

### Pharmacokinetics of favipiravir in patients with SFTS

**Fig 4:**
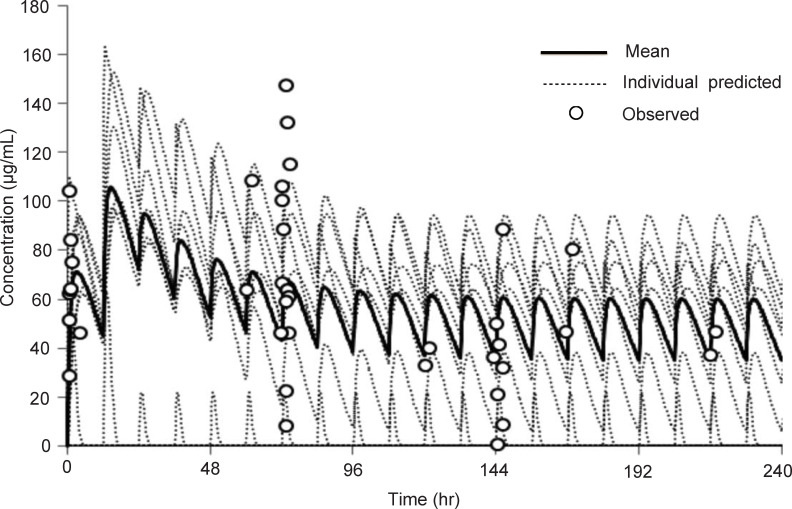
The favipiravir concentration of the patients with SFTS who were administered favipiravir orally. The data were analyzed from the favipiravir concentrations of seven patients. Black and grey dotted lines indicate the mean concentration of favipiravir and predicted concentration of individual patients, respectively. Round circles indicate the concentration of each individual at the designated time points.

Pharmacokinetic analysis was performed using samples from 7 patients (Fig 4). The trough and peak mean concentrations of blood favipiravir were predicted to be about 40 μg/mL and 60 μg/mL, respectively. The blood samples were collected for favipiravir blood concentration measurement only from 12 patients, who participated in the first study period. The blood collection for this purpose was not completed in 2 patients who died and 3 patients because of the patients’ conditions and/or technical issues.

## Discussion

Fifty-five and 69 patients with SFTS were reported to the NIID during the first and the second study periods, respectively [30]. Because SFTS is designated as a notifiable infectious disease, doctors should notify patients with SFTS within 24 hours to the NIID under the regulation of the Infectious Disease Control Law. Therefore, it can be considered that one hundred twenty-four patients reported in total include most of the patents diagnosed with SFTS in Japan. Because all the patients enrolled in this study had also been reported to the NIID, 18.5% of all the patients with SFTS reported to the NIID during the study periods were enrolled in this study and treated with favipiravir.

Favipiravir is converted to its phosphoribosylated metabolites (favipiravir-RMP and favipiravir-RTP) by host cellular kinases, and favipiravir-RTP inhibits the activity of the RNA-dependent RNA polymerase of various RNA viruses [19,31]. The CFR (17.4%) in this study was lower than those reported previously (31% and 27%) in Japan [11,12]. On the other hand, the ribavirin’s efficacy is insufficient and limited [13,16,32–37].

There were no significant differences between the Fatal group and the Non-fatal group in terms of basic patient and clinical characteristics (Table 1) and combination therapies except for mechanical ventilation-based respiratory support (Table 5). A difference in viral genome load was evident at the time of drug initiation (Fig 3 and Table 3). Although the number of the Fatal group and the Non-fatal group were small, the difference was statistically significant. Furthermore, the Fatal group patients received mechanical ventilation-based respiratory support more than the Non-fatal group patients (Table 5). This finding is in accordance with the previous report, in which it was shown that the higher viremia level was a strong risk factor for poor prognosis [13]. The CFRs in SFTS patients with SFTS ≥ 1 × 10^5^ copies/mL of SFTSV genome viremia and those with SFTS < 1 × 10^5^ copies/mL of SFTSV genome viremia in the previous study, in which the association between SFTSV genome levels and outcome was examined in Japan, were 56% (14 of 25 SFTS patients died) and 8% (1 of 12 died), respectively [15]. The decrease in the CFR of the patients treated with favipiravir from those of the previous study in Japan might be achieved by the favipiravir treatment suggesting an efficacy of favipiravir treatment [11,12,15]. It is evident that the results in the present study should not simply be compared with those of these previous studies. This non-comparative design limited our ability to conclude whether favipiravir can reduce the incidence of fatal cases among SFTS patients. However, favipiravir treatment may offer patients with SFTS not only the therapeutic effect in reducing the mortalities, but also that in reducing morbidities. Because SFTSV replicates in the incubation period and the early phase of the disease, the efficacy of favipiravir treatment would be higher, if favipiravir treatment was initiated earlier in early phase of the disease. To have an efficacy, the drug should be initiated as early as possible.

It is written that the peak concentrations of favipiravir in healthy individuals, who were administered orally with favipiravir 1,600 mg twice a day followed by 600 mg/day twice a day for 4 days, was approximately 65μg/mL [38]. It suggests that higher dose of favipiravir should be prescribed for patients with SFTS than that for patients with influenza virus infection. The dosage of favipiravir for patients with SFTS used in this study might be acceptable. Because pharmacokinetic data on favipiravir in patients with SFTS is limited, further study is needed. It is known that gastrointestinal tract symptoms including bloody bowel discharge and bloody vomiting [4,26,39]. Considering the high frequency of central nervous system symptoms and gastrointestinal symptoms in SFTS patients, intravenous administration of favipiravir may be preferable.

The high frequency of hepatic dysfunction as an AE and the observed delay in its improvement are informative when considering the use of favipiravir. As the frequency of liver disorders in SFTS patients is high, it is unclear whether this is merely a side effect of favipiravir.

The CFR of SFTS in Japan could be reduced by approximately 10% by the treatment with favipiravir. There has been a report describing two patients with SFTS treated with favipiravir [40]. In the report, patient #1, a 38-year-old man, and patient #2, a 48-year-old man, were administered favipiravir from day 4 and day 11 from disease onset, respectively. Both patients survived. Because the number of the patients treated with favipiravir is limited in the present study, comparative studies with a larger sample size are warranted to confirm the effects of favipiravir on clinical outcome. The present study suggests that favipiravir may be an effective drug in treatment for patients with SFTS.

## Supporting information

S1 Table Rank classification of clinical symptoms and laboratory findings. For making it available to assess the clinical course of the SFTS patients treated with favipiravir in terms of severity and outcome, severity level of clinical symptoms and laboratory findings were classified in accordance with a pre-specified rank (S1 Table). In brief, the classifications were as follows, Rank 1, normal level; Rank 2, mild level; Rank 3, moderate level; and Rank 4, severe level.
(DOCX)

S2 Table Clinical characteristics of patients on admission. The signs and symptoms of body temperature, headache, Body aches and pains, vomiting, abdominal pain diarrhea, dyspnea, hemorrhagic symptoms, and disorientation of the patients enrolled in the present study on admission was evaluated. The statistical difference was evaluated on each manifestation between the Fatal and Non-fatal groups (S2 Table). The signs and symptoms evident on admission did not significantly differ between the Fatal and Non-fatal groups.
(DOCX)
