# Supplementary material for: A multicenter non-randomized, uncontrolled single arm trial for evaluation of the efficacy and the safety of the treatment with favipiravir for patients with severe fever with thrombocytopenia syndrome
Source: PLoS Negl Trop Dis. 2021 Feb 22;15(2):e0009103. doi: 10.1371/journal.pntd.0009103 (PMC7899362; doi:10.1371/journal.pntd.0009103)
Supplement: S1 Table — For making it available to assess the clinical course of the SFTS patients treated with favipiravir in terms of severity and outcome, severity level of clinical symptoms and laboratory findings were classified in accordance with a pre-specified rank (S1 Table). In brief, the classifications were as follows, Rank 1, normal level; Rank 2, mild level; Rank 3, moderate level; and Rank 4, severe level. (DOCX) [file pntd.0009103.s001.docx]

**S1 Table**

For making it available to assess the clinical course of the SFTS patients treated with favipiravir in terms of severity and outcome, severity level of clinical symptoms and laboratory findings were classified in accordance with a pre-specified rank (S1Table). In brief, the classifications were as follows, Rank 1, normal level; Rank 2, mild level; Rank 3, moderate level; and Rank 4, severe level.

**Table S1. Rank classification of clinical symptoms and laboratory findings.**

| Categories | Rank | | | |
| --- | --- | --- | --- | --- |
|  | 1 (Normal level) | 2 (mild level) | 3 (moderate level) | 4 (severe level) |
| Body temperature (°C) | ≤ 38.0 | 38.0 – ≤ 39.0 | 39.0 – ≤ 40.0 | > 40.0 |
| Headache | Not present | Mild pain | Moderate pain; partially interfere with ADL | Severe pain; impossible to perform ADL |
| Body aches and pains | Not present | Mild pain | Moderate pain; partially interfere with ADL | Severe pain; impossible to perform ADL |
| Vomiting | Not present | 1–2 episodes (separated by 5 min) in 24 hrs | 3–5 episodes (separated by 5 min) in 24 hrs | ≥ 6 episodes (separated by 5 min) in 24 hrs |
| Abdominal pain | Not present | Mild pain | Moderate pain; partially interfere with ADL | Severe pain; impossible to perform ADL |
| Diarrhea | Not present | Increase of < 4 stools per day compared to before onset | Increase of 4–6 stools per day compared to before onset | Increase of ≥ 7 stools per day compared to before onset |
| Respiratory rate indicating dyspnea (beats/min) | 12–24 | 25–34 | 35–49 | ≥ 50 |
| Hemorrhage | Not present | Positive fecal occult blood test | Ulorrhagia | Hematemesis or visible melena |
| Disorientation | Not present | Senselessness with spontaneous eye opening | Eye opening to verbal or pain stimuli | No eye opening to any stimuli |
| Leukopenia (/mm^3^) | ≥ 4,000 | 2,000–> 4,000 | 1,000–2,000 > | 1,000 > |
| Thrombocytopenia (/mm^3^) | > 120,000 | > 80,000–120 000 | > 50,000–80,000 | 50,000 ≥ |
| AST/ALT/LDH | ≤ ULN | > ULN–3.0 × ULN | > 3.0 × ULN–5.0 × ULN | > 5.0 × ULN |
| CK | ≤ ULN | > ULN–5 × ULN | > 2.5 × ULN–5.0 × ULN | > 5.0 × ULN |
| Viral load before initiation of favipiravir treatment (copies/mL) | < UDL | > UDL– < 1.0×10^4^ | ≥1.0×10^4^ – <1.0×10^6^ | ≥1.0×10^6^ |

ADL=activities of daily life, AST=aspartate transaminase, ALT=alanine transaminase, LDH=lactate dehydrogenase, CK=creatine kinase, ULN=upper limit of normal, UDL=under detection limit by qRT-PCR.
