# Supplementary material for: A multicenter non-randomized, uncontrolled single arm trial for evaluation of the efficacy and the safety of the treatment with favipiravir for patients with severe fever with thrombocytopenia syndrome
Source: PLoS Negl Trop Dis. 2021 Feb 22;15(2):e0009103. doi: 10.1371/journal.pntd.0009103 (PMC7899362; doi:10.1371/journal.pntd.0009103)
Supplement: S2 Table — The signs and symptoms of body temperature, headache, Body aches and pains, vomiting, abdominal pain diarrhea, dyspnea, hemorrhagic symptoms, and disorientation of the patients enrolled in the present study on admission was evaluated. The statistical difference was evaluated on each manifestation between the Fatal and Non-fatal groups (S2 Table). The signs and symptoms evident on admission did not significantly differ between the Fatal and Non-fatal groups. (DOCX) [file pntd.0009103.s002.docx]

**S2 Table**

The signs and symptoms of body temperature, headache, Body aches and pains, vomiting, abdominal pain diarrhea, dyspnea, hemorrhagic symptoms, and disorientation of the patients enrolled in the present study on admission was evaluated. The statistical difference was evaluated on each manifestation between the Fatal and Non-fatal groups (S2 Table). The signs and symptoms evident on admission did not significantly differ between the Fatal and Non-fatal groups.

**S2 Table. Clinical characteristics of patients on admission.**

| Clinical manifestations | Numbers of patients evaluated | Overall | Groups | | RR | 95% CI | p-Value* |
| --- | --- | --- | --- | --- | --- | --- | --- |
|  |  |  | Fatal | Non-fatal |  |  |  |
| Body temperature (≥ 38.0 °C) | 23 | 15 (65.2%) | 3/4 (75.0%) | 12/19 (63.2%) | 1.19 | 0.61, 2.30 | 1.00 |
| Headache | 19 | 6 (31.6%) | 2/3 (66.7%) | 4/16 (25.0%) | 2.67 | 0.83, 8.56 | 0.22 |
| Body aches and pains | 18 | 6 (33.3%) | 1/3 (33.3%) | 5/15 (33.3%) | 1.00 | 0.17, 5.77 | 1.00 |
| Vomiting | 21 | 4 (19.0%) | 0/4 (0%) | 4/17 (23.5%) | ·· | ·· | 0.55 |
| Abdominal pain | 18 | 6 (33.3%) | 2/3 (66.7%) | 4/15 (26.7%) | 2.50 | 0.78, 7.97 | 0.25 |
| Diarrhea | 20 | 10 (50.0%) | 3/3 (100.0%) | 7/17 (41.2%) | 2.43 | 1.38, 4.29 | 0.21 |
| Dyspnea | 18 | 5 (27.8%) | 0/2 (0%) | 5/16 (31.3%) | ·· | ·· | 1.00 |
| Hemorrhage | 22 | 4 (18.2%) | 0/4 (0%) | 4/18 (22.2%) | ·· | ·· | 0.55 |
| Disorientation | 21 | 12 (57.1%) | 3/4 (75.0%) | 9/17 (52.9%) | 1.42 | 0.69, 2.92 | 0.60 |

*Fisher’s exact test. RR=risk ratio, CI=confidence interval.
